# Serum Lipoprotein(a) Positively Correlates with Coronary Artery Calcification in Low-Risk Chinese Han Patients: A Study from a Single Center

**DOI:** 10.1371/journal.pone.0071673

**Published:** 2013-08-12

**Authors:** Yibo Jiang, Kai Guo, Mantian Chen, Jun Bao, Chengxing Shen, Yigang Li

**Affiliations:** 1 Department of Cardiology, Xinhua Hospital, Shanghai Jiao Tong University School of Medicine, Shanghai, China; 2 Department of Cardiology, Taixing Hospital, Yangzhou University, Taixing, China; 3 Department of Cardiology, Zhongda Hospital, Southeast University, Nanjing, China; Maastricht University Medical Center, Netherlands

## Abstract

**Background:**

Elevated plasma levels of lipoprotein(a) (Lp(a)) and a higher degree of coronary artery calcification (CAC) are both considered to be risk factors for atherosclerosis. However, previous studies have demonstrated that the relationship between Lp(a) levels and the degree of CAC indicates significant heterogeneity that may be due to varying ethnicities. The purpose of this study was to examine the predictive power of Lp(a) for CAC as measured by multidetector computed tomography (MDCT) in the Han ethnic group of China.

**Methods:**

A total of 1082 subjects were recruited in this study. The patients were divided into four groups: patients without hypertension or diabetes were group 1, patients with hypertension were group 2, patients with diabetes were group 3 and patients with both hypertension and diabetes were group 4. CAC score (CACs), lipid profiles (Lp(a), LDL, HDL, TG, TC), HbA1C, glucose, personal health history and body morphology were measured in all participants. The predictive power of Lp(a) for calcified atherosclerotic plaque was determined by correlations and ordinal logistic regression.

**Results:**

There was no significant difference in the CACs between group 2 and group 3 (z = 1.790, *p = *0.736), and there were significant differences among the other groups. However, there was no significant difference in the total Lp(a) among the 4 groups (χ^2^ = 0.649, *p = *0.885). Only In group 1, Lp(a) was a statistically significant predictor of the presence of calcified coronary plaque using ordinal logistic regression.

**Conclusions:**

Levels of Lp(a) positively correlate with CACs among Chinese Han people who are without diabetes and hypertension, suggesting that Lp(a) may be an important risk factor for the presence of calcified atheromas.

## Introduction

The development of non-invasive imaging techniques, such as multidetector computed tomography (MDCT), allows for visualizing atherosclerotic plaque burden in vivo. Coronary artery calcium scores (CACs) gauged by MDCT have been demonstrated to predict future cardiovascular events in multiple populations [1], [2]. However, the correlation between coronary artery calcium (CAC) and coronary events has not been clearly demonstrated [3]. Additionally, given that the procedures involved in measuring CACs are relatively expensive and require radiation exposure, unselected screening of CAC in subjects may have limited clinical value [4]. As a result, researchers hope to discover biomarkers that are easily measured and that could be used as a substitute for screening individuals with high or low CACs, especially in asymptomatic individuals [5], [6].

Coronary artery disease (CAD) is multifactorial in origin. Many traditional risk factors such as age, gender, smoking, diabetes mellitus, dyslipidemia, and hypertension play an important role in the development of atherosclerotic disorders. Lipoprotein(a) (Lp(a)), an intriguing lipoprotein particle consisting of LDL and apolipoprotein(a) (apo(a)), is also considered to be an important risk factor for the development of CAD.

Previous studies have consistently demonstrated the overall positive associations of Lp(a) levels with CAD [7], [8], [9]. However, controversy still surrounds the role of Lp(a) in predictiing coronary events, especially with respect to gender and race [10], [11]; the mechanisms are still unclear. Thus, Lp(a) has been considered a “conditional risk factor”, in that its utility in risk prediction depends on an individual's background and coexisting conditions such as diabetes [12].

Although Lp(a) is present in atherosclerotic plaques, there have been conflicting data regarding the relationship between Lp(a) and CAC [13]–[16]. Few studies have investigated the relationship between Lp(a) and CAC among the Chinese people. The Han people are the major ethnic group in China among all 56 Chinese ethnic groups, making them a valuable resource for this study. Accordingly, we included 1082 asymptomatic Han participants who had a complete Lp(a) assessment and CAC detection at the time of recruitment and investigated the relationship between Lp(a) and CAC in different subgroups.

## Methods

### Study Participants

All data for this study were recorded for asymptomatic subjects submitted to MDCT scanning at a single center from March 2007 to December 2011. Participants aged 35 to 80 years who identified themselves as Han with an identification card were recruited. To be entered into the database, individuals were required to be asymptomatic for cardiovascular disease and have no prior history of coronary artery disease (CAD) (myocardial infarction, angina, stroke, transient ischemic attack, heart failure, atrial fibrillation, revascularization, valve replacement, pacemaker or defibrillator implantation, or taking nitroglycerin) at the time of enrollment. Other exclusion criteria included pregnancy, active treatment for cancer, serum creatinine>2.5 mg/dl, and the use of cholesterol-lowering medications. Individuals with a triglyceride value of more than 400 mg/dl were unable to be included in the study due to the inability to calculate low-density lipoprotein values using the Friedewald formula [17]. All the data were abstracted from the medical records of the center. All participants gave full written informed consent, and the study protocol was approved by the Committee of Clinical Investigation of Shanghai Jiao Tong University School of Medicine.

Seated blood pressure was measured after 5 min of rest using a random-zero sphygmomanometer, and the average of the last 2 of 3 consecutive measurements was used for analysis. Hypertension was defined as systolic blood pressure (SBP)≥140 mmHg, diastolic blood pressure (DBP)≥90 mmHg, or current antihypertensive therapy. Patients receiving insulin or oral hypoglycemic agents were classified as diabetic. The participants were divided into 4 groups: patients without hypertension or diabetes were group 1, patients with hypertension were group 2, patients with diabetes were group 3 and patients with both hypertension and diabetes were group 4.

Patients with SBP≥140 mmHg or DBP≥90 mmHg upon initial assessment, but who had never been diagnosed with hypertension, underwent further examination. Those patients diagnosed with hypertension upon further examination were placed in group 2, and those who were deemed non-hypertensive were placed in group 1. The same criterion was used regarding assessment for diabetes using blood glucose levels.

A patient described as currently smoking was defined as having smoked a cigarette within the last 30 days. Alcohol use was defined as never, and former or current. Each patient's height and weight were measured, and the body mass index (BMI) was calculated as weight/height^2^ (kg/m^2^).

### Blood Sampling and Lipid Analyses

Fasting venous blood samples were collected into EDTA tubes. Standardized enzymatic methods were used to analysis the serum total cholesterol(TC), high-density lipoprotein cholesterol (HDL-C) and triglyccrides (TG), low-density lipoprotein cholesterol (LDL-C) was calculated using the Friedewald equation. The Lp(a) was analyzed for in freshly isolated sera by electroimmunodiffusion, using the IgG-fraction of specific rabbit anti-Lp(a)-antisera. The plasma concentrations of serum glucose, glycosylated hemoglobin (HbA1C) were also measured.

### Measurement of CAC

MDCT studies were performed using a 64-MDCT scanner. All patients with initial heart rate≥60 beats/min were given an oral beta blocker (metoprolol, 40 mg) to achieve a target heart rate of 50–60 beats/min. Sublingual nitroglycerin was administered just before scanning. A body weight-adjusted volume (0.6–0.7 ml/kg) of iodine contrast (Iopamiron 370, Bayer Healthcare, Berlin, Germany) was administered into the antecubital vein for 10 s followed by 25 ml of saline solution injected at 5.0 ml/s. CT-reconstructed image data were transferred to an offline workstation (Advantage Workstation Ver. 4.2, GE Healthcare) for post-processing and subsequent image analysis. Coronary arteries were assessed using rapid acquisition (100 ms) of 30–40 contiguous slices of 3 mm in thickness during end-diastole using ECG-triggering during a single 30–35 s breath hold. CACs was quantified using the scoring method of Agatston et al [18]. The amount of CAC was classified in the following manner: 0 to 10 (minimal), 10 to 100 (mild), 100 to 400 (moderate), and>400 (extensive) Agatston units [19]. Participants were scanned twice consecutively, and each scan was read by a trained physician independently at a centralized reading center.

### Statistical Analysis

The sample was classified into four groups according to comorbidities of hypertension and diabetes. Comparisons among groups employed analysis of variance (ANOVA) or Kruskal-Wallis tests for continuous variables, and Pearson chi-square tests for categorical variables. The outcome variable for this study was CACs. The primary exposure variable was Lp(a). The covariates included TG, TC, LDL-C, HDL-C, age, gender, BMI, smoking, alcohol, hypertension or diabetes mellitus, HbA1C, SBP, and DBP. The association of Lp(a) with CACs was firstly analyzed with the use of Spearman correlation in each group. Then logistic univariate regression was conducted for Lp(a) and all covariates. Variables with *p*≤0.05 in the univariate analysis were entered into the logistic multivariate regression models. All analyses were performed using SPSS for Windows (version 10.05, SPSS Inc., Chicago, Illinois). A two-tailed *p* value of <0.05 was considered to be statistically significant.

## Results

### 3.1. Characteristics of the Study Sample

A total of 1,082 patients, age 35–80 years, were included. After stratification, there were 302 people in group 1 (male 44.37%), 474 patients in group 2 (male 42.62%), 111 patients in group 3 (n = 111, male 46.85%) and 195 patients in group 4 (n = 195, male 43.08%).

The characteristics stratified by diabetes and hypertension are presented in Table 1. As expected, there were significant differences in glucose, HbA1C, SBP and DBP among the 4 groups. Apart from these variables, age, smoking, BMI, TG, TC, HDL-C and LDL-C also varied significantly among the 4 groups.

**Table 1:** Characteristics of study samples.

variables		Group 1(n = 302)	Group 2(n = 474)	Group 3(n = 111)	Group 4(n = 195)	total(n = 1082)	?^2^	*P*-value
Gender, n%	Male	134(44.4)	202(42.6)	52(46.8)	84(43.1)	472(43.6)	0.757	0.860
Smoker, n%	Yes	100(33.1)	121(25.5)	23(20.7)	61(31.3)	305(28.2)	9.255	0.026
Alcohol, n%	Yes	73(24.2)	116(24.5)	23(20.7)	48(24.6)	260(24.0)	0.757	0.860
Age, n%							21.108	0.012
	35∼	21(7.0)	22(4.6)	5(4.5)	8(4.1)	56(5.2)		
	45∼	59(19.5)	79(16.7)	13(11.7)	25(12.8)	176(16.3)		
	55∼	109(36.1)	137(28.9)	32(28.8)	74(38.0)	352(32.5)		
	65∼	113(37.4)	236(49.8)	61(55.0)	88(45.1)	498(46.0)		
BMI (kg/m^2^), n%	≥25	97(32.1)	152(32.3)	51(45.9)	105(53.8)	406(37.5)	35.160	0.000
Glu (mmol/l), n%	≥6.1	40(13.2)	57(12.0)	80(72.1)	153(78.5)	330(30.5)	420.855	0.000
HbA_1_C(µmol/l),n%	≥6.4	29(9.6)	27(5.7)	100(90.1)	172(88.3)	328(30.3)	694.424	0.000
SBP (mmHg), n%	≥140	21(7.0)	324(68.4)	1(0.9)	139(71.3)	486(44.9)	424.025	0.000
DBP (mmHg), n%	≥90	3(1.0)	70(14.8)	2(0.8)	25(12.8)	100(9.2)	50.055	0.000
Lp(a) (mg/dl), n%	≥30	89(29.5)	134(28.3)	31(27.9)	51(26.2)	305(28.2)	0.649	0.885
LDL-C(mmol/l),%							139.788	0.000
	<2.07	139(46.0)	89(18.8)	24(21.6)	20(10.2)	274(25.3)		
	2.07∼	98(32.5)	184(38.8)	36(32.4)	52(26.7)	368(34.0)		
	3.1∼	65(21.5)	201(42.4)	51(46.0)	123(63.1)	440(40.7)		
HDL-C(mmol/l),n%							105.625	0.000
	<0.83	5(1.6)	25(5.3)	8(7.2)	7(3.6)	45(4.2)		
	0.83∼	169(56.0)	373(78.7)	86(77.5)	168(86.2)	796(73.6)		
	1.96∼	128(42.4)	76(16.0)	17(15.3)	20(10.2)	241(22.2)		
TG (mmol/l), n%							33.428	0.000
	<0.2	0(0.0)	0	0	0	0		
	0.2∼	219(72.5)	296(62.4)	61(55.0)	93(47.7)	669(61.8)		
	2.31∼	83(27.5)	178(37.6)	50(45.0)	102(52.3)	413(38.2)		
TC (mmol/l), n%							68.401	0.000
	<3.36	10(3.3)	8(1.7)	2(1.8)	3(1.5)	23(2.1)		
	3.36∼	282(93.4)	421(88.8)	101(91.0)	142(72.8)	946(87.4)		
	6.46∼	10(3.3)	45(9.5)	8(7.2)	50(25.7)	113(10.5)		

The data were transformed into rank cases or dichotomic variables. BMI = body mass index, Glu = Glucose, HbA1C = glycosylated hemoglobin, SBP = systolic blood pressure, DBP = diastolic blood pressure, Lp(a) = Lipoprotein (a), LDL-C = low density lipoprotein cholesterol, HDL-C = high density lipoprotein cholesterol, TG = triglycerides, TC = total cholesterol. Group 1 included participants without hypertension or diabetes, group 2 included patients with hypertension, group 3 included patients with diabetes and group 4 included patients with both hypertension and diabetes.

### 3.2 CACs and Lp(a) in the 4 Groups

We compared CACs among the 4 groups using the Kruskal-Wallis method. The result was χ^2^ = 75.494, and *p = *0.000, which indicates a significant difference among the 4 groups (Table 2). Additional tests comparing every combination of two groups, except between group 2 and group 3 (z = 1.790, *p = *0.736), all indicated a significant difference among other groups (all, *p<*0.01). The degree of CAC was much higher in the hypertension or diabetes group than that in group 1, and the most severe CAC in general existed within group 4 (hypertension and diabetes). However, there was no significant difference in Lp(a) among the 4 groups (χ^2^ = 0.649, and *p = *0.885).

**Table 2:** Comparison of degree of coronary artery calcium scores (CACs) among groups using the Kruskal-Wallis method.

		Group 1(n = 302)	Group 2(n = 474)	Group 3(n = 111)	Group 4(n = 195)	total(n = 1082)
CACs, n (%)						
	T1	261(86.4)	345(72.8)	71(64.0)	106(54.4)	783(72.4)
	T2	12(4.0)	23(4.9)	7(6.3)	11(5.6)	53(4.9)
	T3	27(8.9)	84(17.7)	27(24.3)	43(22.1)	181(16.7)
	T4	2(0.7)	22(4.6)	6(5.4)	35(17.9)	65(6.0)

T1 = 0∼; T2 = 10∼; T3 = 100∼; T4 = 400∼. Group 1 included participants without hypertension or diabetes, group 2 included patients with hypertension, group 3 included patients with diabetes and group 4 included patients with both hypertension and diabetes.

### 3.3. Association with CAC

The association of Lp(a) with CACs was analyzed with the use of Spearman correlation, the result indicated that Lp(a) significantly correlated with CAC in each group (Table 3), Univariate logistic regression was conducted for all predictor variables using the Kruskal-Wallis method. In group 1, Lp(a), SBP and TC correlated significantly with CACs, whereas TG, TC and BMI were correlated significantly with the CACs in group 2. TC and SBP correlated significantly with CACs in group 4. However, no variable correlated significantly with CACs in group 3 (Table 4). Then multivariate ordinal logistic regression was performed, and predictor variables with a univariate relationship (*p*≤0.05) to CAC were entered into the model, the results of the ordinal logistic regression with Lp(a) as a variable revealed that Lp(a) is a statistically significant predictor for the presence of calcified coronary plaque in group 1 (95% CI 2.499–4.678, *P* = 0.000) (Table 5), and this phenomenon was not observed in the other groups.

**Table 3:** Comparison of the correlation of Lp(a) with CAC using the Sprearman test.

	N	Rs	*P*
Group1	302	0.535	0.000
Group2	474	0.509	0.000
Group3	111	0.479	0.000
Group4	195	0.596	0.000

Group 1 included participants without hypertension or diabetes, group 2 included patients with hypertension, group 3 included patients with diabetes and group 4 included patients with both hypertension and diabetes.

**Table 4:** Predictors for coronary artery calcium scores (CACs) by univariate logistic regression.

	Group 1		Group 2		Group 3		Group 4	
	?^2^	*P*-value	?^2^	*P*-value	?^2^	*P*-value	?^2^	*P*-value
Gender	1.129	0.569	3.529	0.171	1.579	0.454	2.429	0.297
Age	1.559	0.459	1.086	0.581	2. 406	0.300	0.266	0.875
BMI	3.122	0.210	9.617	0.008	0.261	0.878	1.623	0.444
Smoker	0.064	0.968	1.016	0.602	4.134	0.127	12.353	0.002
Alcohol	3.122	0.210	0.315	0.854	0.179	0.914	1.797	0.407
SBP	6.166	0.046	0.316	0.854	5.669	0.059	0.197	0.906
DBP	0.000	1.000	3.573	0.168	0.481	0.786	6.523	0.038
Glu	0.603	0.740	0.781	0.677	0.016	0.992	0.026	0.987
HbA_1_C	2.095	0.351	0.413	0.813	1.262	0.532	1.543	0.462
Lp(a)	10.450	0.005	3.497	0.174	2.902	0.234	5.480	0.065
LDL-C	1.875	0.392	2.327	0.312	0.862	0.650	5.267	0.072
HDL-C	0.603	0.740	5.530	0.063	2.129	0.345	1.107	0.575
TG	2.213	0.315	6.129	0.047	1.839	0.399	2.751	0.253
TC	7.817	0.020	15.687	0.000	2.481	0.289	23.096	0.000

BMI = body mass index, Glu = Glucose, HbA1C = glycosylated hemoglobin, SBP = systolic blood pressure, DBP = diastolic blood pressure, Lp (a) = Lipoprotein (a), LDL-C = low density lipoprotein cholesterol, HDL-C = high density lipoprotein cholesterol, TG = triglycerides, TC = total cholesterol. Group 1 included participants without hypertension or diabetes, group 2 included patients with hypertension, group 3 included patients with diabetes and group 4 included patients with both hypertension and diabetes.

**Table 5:** Predictors for coronary artery calcium scores (CACs) by ordinal logistic regression.

Group	Predictor	Estimate	Std.Error	Wald	95% CI	*p*-value
Group 1	SBP	2.660E-02	0.691	0.001	−1.328 to1.381	0.969
	LP(a)	3.588	0.556	41.567	2.497–4.678	0.000
	TC	2.188	0.691	10.034	0.834–3.542	0.002
Group 2	TC	1.906	0.356	28.693	1.209–2.604	0.000
	TG	2.798	0.288	94.597	2.234–3.362	0.000
	BMI	1.406	0.269	27.245	0.878–1.934	0.000
Group 3	DBP	0.399	0.443	0.813	−0.469 to1.268	0.367
	TC	3.730	0.440	72.022	2.869–4.592	0.000

CI = Confidence interval. Group 1 included participants without hypertension or diabetes, group 2 included patients with hypertension, group 3 included patients with diabetes.

## Discussion

To our knowledge, this is the first study to investigate the relationship between Lp(a) and CAC in the Han ethnic group of China. The results of the current study indicate that Lp(a) is significantly correlated with and predictive of prevalent coronary calcification in the asymptomatic Chinese Han people who are without diabetes and hypertension.

Recent advances in MDCT enabled the detection of calcified coronary atherosclerotic plaque and non-calcified coronary atherosclerotic plaque, which was consistent with intravascular ultrasound [19], [20], [21]. Various studies have indicated that CAC scoring detected coronary atherosclerosis and improved the risk stratification of individuals beyond traditional cardiovascular risk factors [1], [2]. Guidelines recently recommended the use of CACs for selected subjects for predicting a coronary heart disease event [22].

Numerous publications have reported that ethnicity affects the presence and severity of CAC independent of atherosclerotic risk factors. Several large epidemiologic studies have demonstrated that white race is a strong predictor of CAC relative to African-Americans men of similar age [14], [23], [24]; however, the relationship in women appears to be less robust, possibly due to gender differences in metabolic risk factors. Kim et al. discovered that CAC is much lower in the Korean population in general than that among Caucasians [25]. Undoubtedly, many other factors, including gender, age, hypertension, obesity, and diabetes, affected the presence and the degree of CAC [26]. Koulaouzidis et al. [27] studied 388 individuals with a CACs of zero and determined that the average time of new CAC development was 4.2±1.1 years. Individuals with CAC progression presented ahigher incidence of hypertension, diabetes mellitus and hypercholesterolemia and a higher frequency of male gender than those without CAC changes. Viera et al. [28] examined 239 young participants and discovered a U-shaped relationship between the systolic BP dipping ratio and future CAC that persisted after adjustment for multiple potential confounders. Kramer and colleagues [29] investigated 338 older adults without known heart disease. They discovered that blood pressure levels and fasting plasma glucose were better independent determinants of CAC progression than metabolic syndrome itself.

The Chinese population also has significantly different genetic and environmental backgrounds from other ethnicities. China has 56 ethnic groups; their genetic backgrounds, customs, culture, and food consumption are strikingly different. Wu et al. reported that the incidence and mortality rates for CAD were obviously higher in the northern regions compared with those in the southern regions in China [30]. The reason for the differences is most likely due to a greater incidence of risk factors such as hypertension, obesity, current smoking, dyslipidemia and diabetes in the northern regions compared with the southern regions [31]. The Han ethnic group is the major group in China, making it a very valuable resource for this study. We could not compare these results directly with the findings of others because we did not enroll individuals of other ethnic groups in this study, and certainly, the characteristics among subjects in these studies were very different.

The results of our study indicated that the degree of CAC was much greater in patients with hypertension or diabetes than others. Additionally, the most severe coronary artery calcium in general existed in patients with hypertension and diabetes, which is consistent with the option that the presence and progression of CAC correlated significantly with hypertension and diabetes [27], [29].

Although CACs can provide an estimate of the total coronary atherosclerotic plaque burden, the correlation between CAC and coronary events may still be suboptimal [3], and unselected screening of CAC in subjects may have limited clinical value [4]. A possible reason for this finding is that the procedures involved in measuring CACs are relatively expensive and require radiation exposure. Discovering biomarkers associated with cardiovascular risk could be helpful in discerning individuals with high or low CACs, especially in asymptomatic individuals [5], [6].

Lp(a) is a plasma lipoprotein that consists of an LDL molecule and apolipoprotein(a) (apo(a)), which contains multiple repeats resembling plasminogen kringle 4. Lp(a) has been detected in atherosclerotic plaques [32] and endothelial damage. Numerous studies have consistently demonstrated the overall positive correlation of Lp(a) levels with CAD [7], [8], [9]. In addition, many studies have attempted to determine the value of Lp(a) to CAC; however, the relationship between Lp(a) and CACs was conflicting. Qasim et al. investigated the relationship between Lp(a) and CACs in type 2 diabetic subjects. A total of 1,299 subjects with type 2 diabetes and 860 without diabetes were enrolled. The association of Lp(a) with CAC remained significant [Tobit regression ratio 2.76 (95% CI 1.73–4.40), *p*<0.001] in diabetic women. In contrast, Lp(a) was not associated with CAC in diabetic men nor in non-diabetic men and women [12]. Greif et al. examined 1560 European patients (1123 men, age 59.3±20.8 years) with typical or atypical chest pain and deemed Lp(a) as an independent risk factor for CAC (*p*<0.001) [33]. Sharma et al. compared 103 consecutive patients in the USA with 104 consecutive patients in Indonesia. They determined that Lp(a) remained an independent predictor of CAC with an odds ratio of 4.97 (95% confidence interval, 1.56–15.88; *p*<0.0001) in Southeast Asians but not in Caucasians [34]. Cassidy et al. examined 616 asymptomatic Caucasians and discovered that Lp(a) alone was a significant predictor for CAC in women (*P = *0.04) [13]. Lee et al. examined 1000 young (age 40–45, 19.4% black) healthy individuals and also observed a positive association [14]. However, in the GENOA study, 756 Caucasians with hypertension were enrolled (59% women, of whom 14% were diabetic), and no relationship was discovered between Lp(a) and CACs [15]. Similarly, in the multi-ethnic Dallas Heart Study, 761 blacks and 527 whites (8% diabetic, half women) were enrolled, and there was no clear relationship between Lp(a) and CACs [16].

In our study, Lp(a) significantly correlated with CAC in each group after spearman test at first. However, after univariate logistic regression, In group 1, Lp(a), SBP and TC correlated significantly with CACs, whereas TG, TC and BMI were correlated significantly with the CACs in group 2, TC and SBP correlated significantly with CACs in group 4, and no variable correlated significantly with CACs in group 3. After the multivariate ordinal logistic regression was performed, the results revealed that Lp(a) is a statistically significant predictor for the presence of calcified coronary plaque in group 1, Lp(a) was associated with CACs in asymptomatic Chinese people without diabetes and hypertension, which represented those healthy populations or low risk patients, and we have not observed the same result in other groups. The exactly mechanism for this difference is still uncertain. The most important reason perhaps is that many patients received the drug treatment in group 2, group 3, group 4, and not in group 1, another possible reason is the number of the patients included in this study is still small. And ethnic variation perhaps should be taken into account when considering the use of Lp(a) measurements in risk assessment. Additionally, the discrepancy may be partly due to the differences in socioeconomic status, access to care, and lifestyle changes. Further research is required to determine factors influencing the differences.

This study has several limitations. First, it was conducted at one center, limiting the generalization of our finding to all Han ethnic in other regions in China; therefore, conclusions about causality cannot be made. Second, many patients choosing the traditional Chinese medicine to treat the hypertension or diabetes, and the diversity of these drugs and the complexity of the compositions made it difficult to do the relative statistics. Therefore, it is possible that our results are partially biased by some of these unmeasured factors. Finally, because the participants were divided into 4 groups for this study, there were relatively small numbers in each subgroup for analysis.

In conclusion, our data suggested that an increased Lp(a) concentration correlated significantly with CAC only in asymptomatic and low-risk Han people. These findings limit the use of Lp(a) as a biomarker for the evaluation of risk for cardiovascular disease in all Chinese ethnics. However, a single measure of Lp(a) concentration to predict CAC in those low-risk people with extreme values may have some utility. Further study in larger, multicenter populations is needed to verify this finding.
